# Motion Plan Changes Predictably in Dyadic Reaching

**DOI:** 10.1371/journal.pone.0167314

**Published:** 2016-12-02

**Authors:** Atsushi Takagi, Niek Beckers, Etienne Burdet

**Affiliations:** 1 Department of Bioengineering, Imperial College of Science, Technology and Medicine, London, United Kingdom; 2 Department of Biomechanical Engineering, University of Twente, Enschede, The Netherlands; Tokyo Daigaku, JAPAN

## Abstract

Parents can effortlessly assist their child to walk, but the mechanism behind such physical coordination is still unknown. Studies have suggested that physical coordination is achieved by interacting humans who update their movement or motion plan in response to the partner’s behaviour. Here, we tested rigidly coupled pairs in a joint reaching task to observe such changes in the partners’ motion plans. However, the joint reaching movements were surprisingly consistent across different trials. A computational model that we developed demonstrated that the two partners had a distinct motion plan, which did not change with time. These results suggest that rigidly coupled pairs accomplish joint reaching movements by relying on a pre-programmed motion plan that is independent of the partner’s behaviour.

## Introduction

From a parent coordinating movements to help a child learn to walk, to a therapist supporting a patient during recovery of their motor functions after injury or disease, we rely on physical interaction while performing tasks with a common goal. Despite its importance, physical coordination has only recently been investigated [1–9]. These studies use a variety of metrics such as distance from a goal [1,4,5], ad-hoc roles [2,6,7], magnitude of interaction force from the physical coupling [3], the energy exchanged between partners [8] and dominance measures [9] to quantitatively analyse physical interaction. Importantly, these studies measured only the outcome of physical interaction and could only speculate as to the cause of these outcomes, which explains why we still have limited understanding of how two people complete a common task.

To understand how pairs or dyads physically *coordinate* motion behaviours, which we define as the outcome of partners who change their movement or motion plan dependent on the partner’s actions, we examined physically connected partners whose task was to reach a common target from the same initial position. Since the reaching movement is discrete, we can measure trial-by-trial change in the kinematic trajectory and the interaction force. To interpret these changes in kinematics and force, we need a computational model of two coupled partners control. Without a model, the kinematics and interaction force alone cannot differentiate between one partner attempting to move faster or the other to move slower [10]. However, a computational model can resolve this redundancy as each dyad simulated by two coupled controllers yields a unique trajectory and interaction force pattern that can be compared with data. By using our experimental protocol and computational model together, we address the limitations of a previous study that purported that the interaction force is used to negotiate changes in motion plan, but could not support this claim [2].

If coupled partners do physically coordinate the interaction force sensed through *haptics*, the sensory modality of touch and proprioception, in what manner would they change their motion plan? Reaching movements in humans have been shown to minimize error, e.g. distance of the hand from a target, and effort [11]. This can be modelled as the minimization of a cost function, which yields a motion plan. We hypothesize that dyads physically coordinate by reducing effort in the form of interaction force that does not contribute to the movement [7]. However, other studies have suggested that the interaction force is critical to physical coordination [2,3]. Do partners update their motion plan to reduce interaction force in order to conserve effort, or increase it to improve coordination? To answer this question, we also tested dyads who were constrained to produce a constant interaction force prior to the initiation of the reaching movement. If partners coordinate by minimizing force, their motion plans should update trial-by-trial to decrease this interaction force. The results suggest that the two partners use distinct motion plans unmodified throughout the trials.

## Methods

### Participants

Sixteen healthy participants (eight males, eight females; mean age: 25.9 years, age range: 22–33 years) were recruited in pairs to form eight *dyads*. Pairs were matched on sex to avoid any large differences in physical strength. All participants were right-handed, as assessed using the Edinburgh Handedness Inventory [12]. All participants gave informed consent prior to participation, and the experiment was conducted in accordance with the Declaration of Helsinki and approved by the Imperial College Research Ethics Committee.

### Apparatus

**Fig 1:**
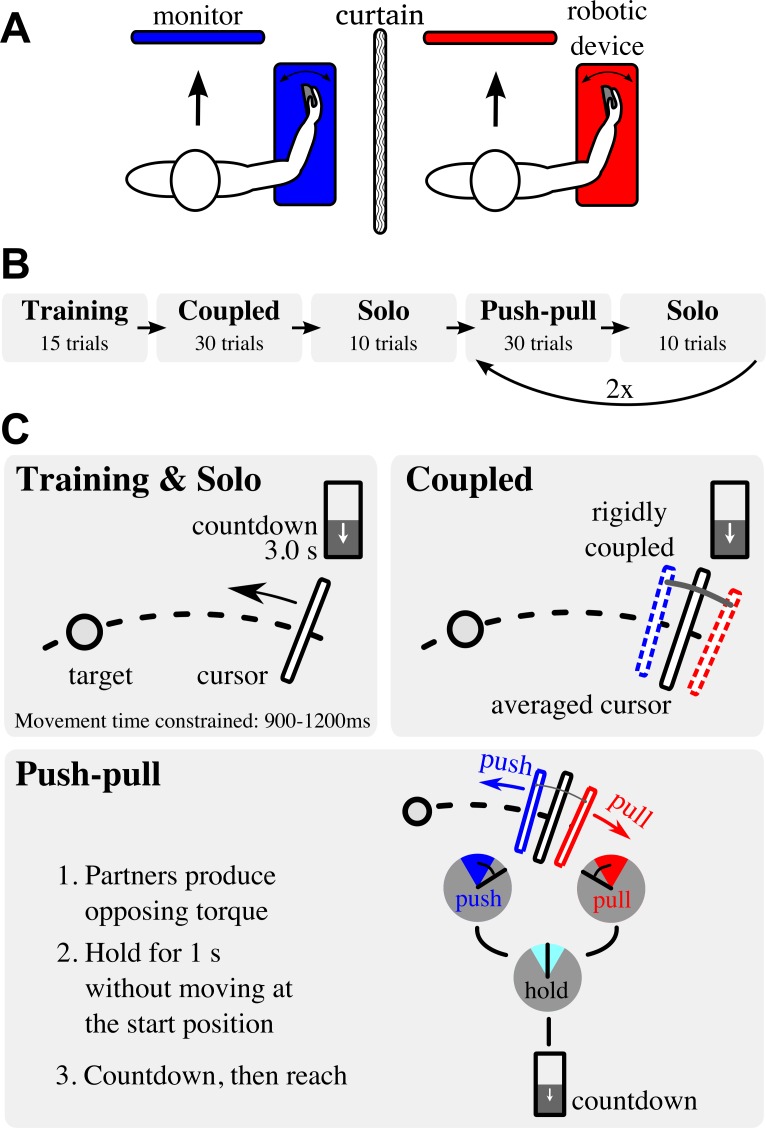
Dyadic reaching apparatus and protocol. (A) Schematic of the dual-wrist interface setup. (B) The experiment was split up into three types of blocks: training and solo blocks, a coupled block and push-pull blocks. Dyads were alternately disconnected (training and solo blocks) and connected (coupled and push-pull blocks). After the first push-pull block and subsequent solo block, dyads performed another push-pull block and solo block. (C) In the training and solo blocks, partners are disconnected and reach the target alone. Feedback was given to the subject if the movement is faster than 900 ms or slower than 1200 ms. In the coupled and push-pull blocks the partners were rigidly coupled. In the coupled block, partners were rigidly coupled and moved the averaged cursor to the target. In the push-pull block, dyads were constrained to produce a torque of 0.7 Nm prior to the reaching movement. One partner pushed towards the target and the other pulled away; once the initial opposing torque was balanced and the average cursor position at 0 degrees, the 3 second countdown was initiated. Partners switched the pushing and pulling instructions in the second push-pull block. In all blocks, a 3 second countdown was used to initiate the movement.

The experiments were performed in dyads using the Hi5 dual one degree-of-freedom wrist robotic interface described in [13] (see Fig 1A). Each participant placed his or her right wrist in the Hi5 robotic interface, which allowed flexion and extension of the wrist. The wrist movement controlled a cursor on one’s own computer display [13] (Fig 1B). The robotic interface can generate a stiff connection between both wrist interfaces with a torsional stiffness of 23 Nm/rad, which is equivalent to a linear stiffness of approximately 2300 N/m when assuming the pivot arm is 0.1 m from the centre of the wrist (centre of rotation) to the middle of the palm. Participants were separated by a heavy curtain, which prohibited them from seeing each other’s movements, displays, and eliminated social interaction.

### Experiment protocol

In each trial, participants made point-to-point reaching movements using the dual robotic interface with wrist flexion/extension. The movement’s start and target positions were fixed at 10 degrees extension and 30 degrees flexion, respectively. To synchronise the movement start, participants initiated their movement after a 3 second countdown, which was shown on the display (see Fig 1C). The trial ended when the cursor was held in the target for 0.5 seconds.

Dyads performed 135 trials in seven blocks in which their wrists were alternately not connected (one *training* and three *solo blocks*) and connected (one *coupled* and two *push-pull blocks*), see Fig 1B.

We constrained movement time during the training and solo blocks between 900 ms and 1200 ms for two reasons: (1) to allow for more time for interaction to occur compared to previous studies that showed variable movement times smaller than 800 ms [2,3] and (2) to return partners to their baseline reaching movement such that the dyads started each coupled and push-pull block from the same condition. For this purpose, participants received feedback on the display using the messages “too fast” or “too slow” after a movement with duration smaller than 900 ms or larger than 1200 ms, respectively. Movement time was not constrained during the coupled and push-pull blocks since our aim is to investigate changes in coordination due to interaction forces, not due to changes in movement behaviour to adjust for too short or too long movement durations.

Dyads first performed a *training block* of 15 trials to familiarize themselves with the interface and the reaching task, including the countdown and movement time constraint. During this training block, participants’ wrists were not connected such that the participants independently reached towards the target (training & solo panel of Fig 1C).

After training, the partners experienced a *coupled block* of 30 trials, where their wrists were rigidly coupled by the computer-generated rigid connection. Instructions and procedure per trial in the coupled block were the same as in the training block; participants had to make reaching movements after a 3 second countdown (coupled panel of Fig 1C). Although the connection was very stiff, the connection could still result in very small differences in cursor position between the partners. Therefore, the average wrist position was displayed as the common cursor. Through the rigid coupling, participants could exert torques onto each other.

Following the coupled block, participants performed a *solo block* of 10 trials, which was used to allow the participants to return to their baseline reaching behaviour.

Dyads then experienced two *push-pull blocks* (30 trials per block) in which each reaching movement started with an initial opposing torque while being rigidly coupled (see Fig 1C). Partners were instructed to produce an initial constant torque of 0.7 Nm whilst remaining stationary at the starting position prior to the movement onset only. Once the torque was held for 1 second, the 3 second countdown was initiated and partners were instructed to reach to the target at the end of the countdown. During the countdown, dyads had to maintain the initial opposing torque and remain stationary at the starting position. No further instructions were given concerning the opposing torque during the reaching movement itself. In one push-pull block, one partner was assigned to *push towards* the target (i.e. produce a positive torque) and the other to *pull away* from it (i.e. produce a negative torque). In the next push-pull block, the instructions of pushing and pulling were switched. The order of which partner pushed towards the target was counterbalanced and randomly assigned to dyads. Each push-pull block was followed by a solo block.

### Data analysis

Trajectory and interaction torque data was recorded at 100 Hz. Data was recorded from the start of the countdown until the cursor was in the target for 0.5 s. All trajectory and torque data were aligned on the start of a trial, which was defined as the time the wrist velocity exceeded 5 degrees/s. Data was then truncated after 1.2 s, which was the slowest permissible movement time as constrained during the solo trials. The trajectory and torque patterns of all trials were resampled to 256 time samples. Trajectory and torque patterns were filtered using a zero-phase sixth-order low-pass Butterworth filter with a cut-off frequency of 6 Hz.

Changes in physical coordination throughout the coupled and both push-pull blocks were analysed per block by comparing the trial-by-trial changes in trajectory and torque patterns within each block. In addition, since we observed dyad-specific torque patterns, this analysis was performed for each dyad separately. To compare the trajectory and torque patterns we used wavelet-based ANOVA following the method described by McKay et al. (see [14] for a detailed description). By performing the time-series analysis in the wavelet domain, statistical power is increased relatively to time domain ANOVA, since differences between curves tend to be represented by a few wavelets and hence results in fewer comparisons. Furthermore, this method does not sacrifice temporal resolution which occurs when dividing the trajectory or torque patterns into time windows [14].

All trajectory and torque patterns were transformed to the wavelet domain using the MATLAB wavelet toolbox (third-order coiflets, decomposition level 4). This resulted in 256 wavelet coefficients for the trajectory and torque patterns for each trial. We grouped the 30 trials per coupled or push-pull block into six bins of five trials each in chronological order (e.g. bin 1: trials 1–5, bin 2: trials 6–10, etc.). By performing a wavelet-based ANOVA on the binned data with bin as single factor, we can test whether the trajectory and torque patterns change across bins and hence throughout each connected block. Significant differences in trajectory or torque patterns between bins could indicate changes in physical coordination. A fixed-effect single-factor ANOVA model with bin as factor at a significance level of 0.05 was performed for each wavelet coefficient across bins, resulting in 256 *F* tests per block and per dyad. Post hoc multiple pairwise comparisons between bins using Scheffé’s method were performed on the wavelet coefficients corresponding to the significant *F* tests. Post hoc tests were performed at significance level of 0.05 which was Bonferroni-adjusted for the number of significant initial *F* tests. Significantly different wavelet coefficients were transformed back to the time domain for visualisation and analysis. This results in time-domain curves describing the mean differences of trajectory and torque patterns and temporal location of these differences between all combinations of bins, per connected block and per dyad [14]. Point-wise ANOVA were also performed in the time domain to corroborate the findings of the wavelet-based ANOVA. S3 Fig shows an example of the mean difference curves as a result of the wavelet-domain and time-domain analysis for the coupled block of dyad VIII. All significant differences and temporal location of the differences in torque curves are summarized in S1 Table for all connected blocks and all dyads.

### Model of dyadic reaching

A participant reaching in one dimension using their wrist with state

$$x={\left[\theta ,\dot{\theta },\tau ,f\right]}^{T},$$ (1)

composed of the angle, angular velocity, torque and an auxiliary variable, evolves in discrete time through the state space model

$${x_{k+1}={\mathrm{Ax}}_{k}+Bu_{k}+C(x}_{k}^{p}-x_{k})$$ (2)

where $x_{k}^{p}$ is the state of the partner, *k* the time index,

$$A\equiv \left[\begin{array}{cccc} 1 & \Delta t & 0 & 0\\ 0 & 1 & \Delta t/I & 0\\ 0 & 0 & 1-\Delta t/\tau_{u} & \Delta t/\tau_{u}\\ 0 & 0 & 0 & 1-\Delta t/\tau_{u} \end{array}\right],\;B\equiv \left[\begin{array}{c} 0\\ 0\\ 0\\ \Delta t/\tau_{u} \end{array}\right],\;C\equiv \left[\begin{array}{cccc} 0 & 0 & 0 & 0\\ K\;\Delta t/I & D\;\Delta t/I & 0 & 0\\ 0 & 0 & 0 & 0\\ 0 & 0 & 0 & 0 \end{array}\right]$$ (3)

with the moment of inertia of the wrist *I*, time step Δ*t* and the stiffness and damping of the spring *K* and *D* that connects partners. The musculoskeletal system of the wrist is modelled using a second-order muscle-like filter with a time constant of *τ*_*u*_ = 40ms [15]. The connection matrix **C** ≡ 0 if the partners are disconnected, as in the solo block. The motor command *u*_*k*_ sent to the wrist is

$$u_{k}=-L(x_{k}-t)$$ (4)

where the target vector ***t*** is

$$t={\left[\theta_{t},\;0,\;0,\;0\right]}^{T}$$ (5)

during reaching, with *θ*_*t*_ is the target angle of the movement. In addition, for the push-pull blocks, the target vector prior to the reaching movement in the push-pull blocks is

$$t={\left[\theta_{0},\;0,\;\tau_{0},\;0\right]}^{T}$$ (6)

where *θ*_0_ and *τ*_0_ are the initial angle and the interaction torque imposed prior to the movement in the push-pull blocks, respectively.

The controller gain **L** is computed by minimising the cost

$$\sum_{k=0}^{\infty }x_{k}^{T}Qx_{k}+u_{k}Ru_{k}$$ (7)

where the matrix **Q** is positive semi-definite and *R* is positive. The solution of this infinite horizon linear quadratic regulator (LQR) is [16]

$$S=Q+A^{T}[S-\mathrm{SB}{\left(R+B^{T}\mathrm{SB}\right)}^{T}B^{T}S]A$$ (8)

and the iterative solution of Eq 8 yields the optimal gain

$$L={\left(R+B^{T}\mathrm{SB}\right)}^{T}B^{T}\mathrm{SA}.$$ (9)

A pair of the human-like controllers were simulated in parallel, only interacting through the connection matrix **C**. Thus, each partner was affected by the interaction forces alone, and did not plan a joint movement with another partner, yielding *co-activity* [17].

We assume that participants know the state of their own wrist with fidelity. For all simulations, the parameters were set to

$$K=0.4\mathrm{Nm}/\deg,\;D=0.05\mathrm{Nms}/\deg,\;R=0.5,\;dt=0.01s,\;I=0.1\mathrm{kg}m^{2}$$ (10)

*K* is from the experiment and damping *D* is added to model the natural damping properties of the wrist. The inertia *I* and the control cost *R* were set to match the average speed and torque observed in the data such that the motion plan identification (described in the next section) converged within reasonable time for each dyad. The initial and target angles and initial opposing torque were set to

$$\theta_{0}=-10\;\deg,$$ (11)

$$\theta_{t}=30\;\deg,$$ (12)

$$\tau_{0}=+0.7\;\mathrm{or}\;-0.7\;\mathrm{Nm}$$ (13)

as in the experiment.

### Identifying the motion plan

To identify the parameters of the cost function **Q** (in Eq 7) and the final target angle of each partner (Eqs 5 and 6), we conducted a tree-search of the 6 parameters Q_1_(1,1), Q_1_ (2,2), *θ*_1_ and Q_1_(1,1), Q_2_(2,2), *θ*_2_ to identify their values that minimized the normalized squared distance from the trajectory and force pattern in the data

$$M=\frac{1}{2L}\sum_{i=0}^{L}\frac{{\left({\bar{\theta }}_{i}-\theta_{i}\right)}^{2}}{\sigma_{\theta_{i}}^{2}}+\frac{{\left({\bar{\tau }}_{i}-\tau_{i}\right)}^{2}}{\sigma_{\tau_{i}}^{2}}$$ (14)

where *L* is the length of data, $\bar{\theta }$ is the mean angle of the data over all trials in one block, $\bar{\tau }$ is the mean torque in one block, $\sigma_{\theta_{i}}^{2}$ and $\sigma_{\tau_{i}}^{2}$ are the variance of the angle and torque along the trajectory, and *θ* and *τ* are the angle and torque in the simulation. The parameters were initialized with the values

Q=diag(2,0.1,0,0),θ=30° (15)

which were incrementally altered in step sizes of δQ_pos_ = 1, δQ_vel_ = 0.05 and δ*θ* = 1° (for position cost, velocity cost and final target angle) to minimize the magnitude of the metric in Eq 14. The algorithm goes as follows: first, we simulated the 64 permutations of Q_1_(1,1) ± δQ_pos_, Q_1_(2,2) ± δQ_vel_, *θ*_1_ ± δ*θ*, Q_2_(1,1) ± δQ_pos_, Q_2_(2,2) ± δQ_vel_ and *θ*_2_ ± δ*θ*. Out of these permutations, the parameters that minimized the metric of Eq 14 was selected as the starting point again, and the process was repeated for 200 iterations for each dyad in the coupled and push-pull blocks. We then compared the relative changes in the position and velocity cost terms in the cost matrix **Q** between the coupled and push-pull blocks to examine any consistent change due to the torque prior to movement onset.

## Results

**Fig 2:**
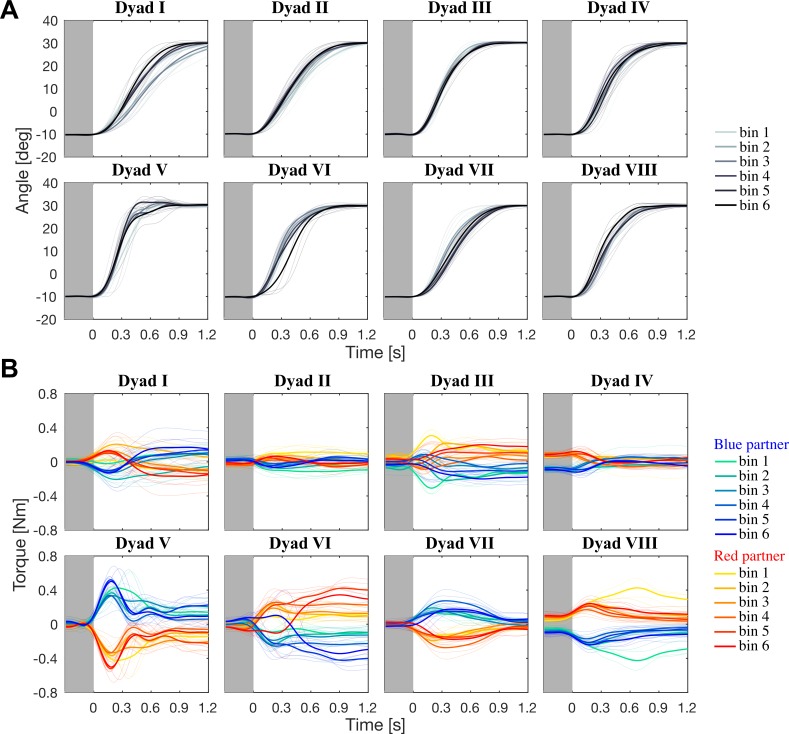
Trajectory and torque data for the coupled block. (A) Trajectories of the average cursor for all dyads in the coupled block. The bold lines indicate the average trajectory of each bin; the thin lines show the individual trials. (B) Torque from all eight dyads, where each bold trace corresponds to the average of each bin; the bins progress chronologically from green to blue for the blue partner and from yellow to red for the red partner. With the exception of dyad VIII, no consistent trial-by-trial change in the torque was observed within dyads. Different dyads displayed specific torque patterns.

We first examined the trajectories and interaction torques from the coupled block. The trajectories of the average cursor from all binned trials in the coupled block are shown in Fig 2A, where each bin consists of five trials. The trajectories were different between dyads, but a statistical analysis showed no significant differences between the binned trials within dyads (for all *F* tests, *p* > 0.05, see Methods for a description of the analysis). Fig 2B shows the torque patterns from all dyads in the coupled block. Similar to the kinematics, we observed dyad-specific torque patterns. The partners in dyad II, for example, have specialised into pure acceleration (blue partner) and deceleration (red partner) of the coupled movement. Some statistical differences were found between within-dyad binned torque patterns; dyad III showed a difference in torque between bins 2 and 3 for only a small time window (0.38 to 0.45 s; one significant *F* test, *p* < 0.05), see S1 Table. However, we observed *no consistent trial-by-trial changes* in the torque patterns for any dyad in the coupled block, with the exception of dyad VIII. Dyad VIII showed significantly higher torques in bin 1 compared to bins 2 to 6 (see S3–S11 Figs and S1 Table for details). Although the torques in bin 1 were higher, the torques in the other bins were consistent and showed no significant differences between bins. These data suggest that dyad VIII changed their motion plan after bin 1, but did not coordinate during bins 2 to 6, i.e. they did not change their motion plan to reduce effort.

### Dyadic reaching with torque at movement onset

**Fig 3:**
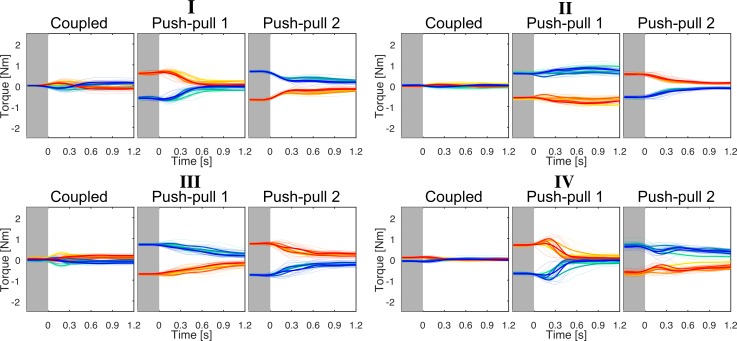
Dyadic reaching with initial opposing torque prior to movement. Torque from dyads I–IV in the coupled and both push-pull blocks; each bold trace is the average trajectory of each bin. In all dyadic reaching blocks, the torque was unchanging between trials within each block.

Next, we examined the torque patterns of all dyads in the push-pull blocks. The representative dyads I–IV are shown in Fig 3, while dyads V–VIII are plot in S2 Fig. It appears that the torque patterns are unchanging from one trial to the next in each push-pull block. The wavelet-based ANOVA did reveal some significant differences in between bins for dyads I, VI, VII and VIII, see S1 Table. For instance, dyad I showed differences in torque between bin 1 and bins 5 and 6, but only briefly, and dyad VII’s torque pattern in bin 2 was different compared to bins 4 and 5 in the last 0.5 s of the reaching movement (see S1 Table for details). Importantly, the significant differences were not consistent across the push-pull bins for all dyads and across both push-pull blocks.

There are three noticeable outcomes in the push-pull condition. First, partners in the same dyad do not have identical torque patterns when switching between pushing and pulling roles. Second, the torque pattern predominantly remains in the same direction throughout the whole movement, i.e. a partner constrained to push at movement onset keeps pushing and vice versa. Third, the torque at movement onset caused all dyads, in at least one push-pull block, to end the movement with constant (e.g. non-zero) opposing torque (paired-sampled *t*-test on the opposing torque at *t =* 1.2 s, *t*(15) *=* 46.80, *p* < 0.001). The constant opposing torque at movement end is possible due to the redundancy of the task, but is functionally superfluous and energy inefficient. Partners could have updated their motion plans between trials to reduce this superfluous torque, but they did not.

### Simulation of dyadic reaching

Why do the trajectory and torque patterns remain relatively stable from trial-to-trial within each block? Why is the torque pattern dyad-specific, and how can the same dyad exhibit different trajectories and torque patterns between the coupled and push-pull blocks? To address these questions, we developed a computational model of dyadic reaching. The experimental results suggest that partners do not update their motion plan from trial-to-trial, but may have differing motion plans in each block. Thus, we assume that the torque patterns are a by-product of two rigidly coupled participants who planned their motion independently, corresponding to the mechanism of *co-activity* [17]. Each participant was modelled as a controller with muscle-like dynamics that minimized the difference in position and velocity between the cursor and target using a linear quadratic regulator [18]. Dyadic reaching was simulated with two of these controller agents who planned and executed their movements independently with movement affected by the torque of the rigid coupling.

**Fig 4:**
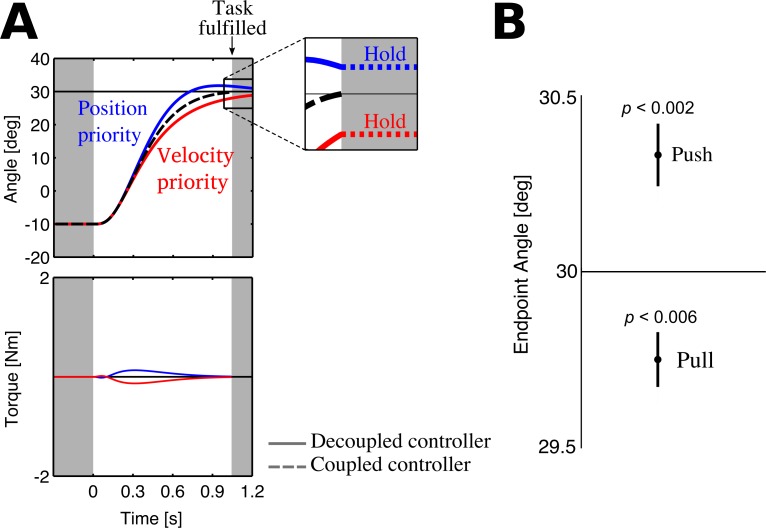
Position and velocity priority in the motion plan explains endpoint bias. (A) The (decoupled) blue controller prioritises position, which causes it to overshoot the target. The (decoupled) red controller prioritises velocity such that it converges to the target without any overshoot. When the position-priority and velocity-priority controllers are coupled (dashed black trace), a force pattern is observed (dashed blue and red traces). The only manner in which the controllers would end the movement with constant torque is if they decide to hold their position once the reach is fulfilled. (B) Endpoint bias at the end of the reaching movement from all 16 partners. Partners, at movement onset, who pushed towards the target overshot it, and those who pulled away undershot it.

As the trajectory and torque pattern in different block conditions was different for each dyad, partners may have had different motion plans between block conditions that did not change within each block. To identify the motion plans in each block for each partner, we modified each partner’s state cost matrix **Q**, which has two diagonal terms that prioritise the difference between the current and desired position and velocity (see Methods for details). How do these two terms in the state cost matrix affect the motion plan? A *position-priority controller*, with a large term in **Q**(1,1) and a small term in **Q**(2,2), will aggressively reach to the target, resulting in large overshoot (blue decoupled controller in Fig 4A). A *velocity-priority controller*, with a small term in **Q**(1,1) and a large term in **Q**(2,2), will slowly converge to the target without overshooting (red decoupled controller in Fig 4A).

When these two controllers, one position-priority and the other velocity-priority, are rigidly coupled, an interaction torque pattern is observed (see dashed torque traces in Fig 4A) that appears similar to dyad II’s coupled reaching (Fig 3). However, the simulated torque pattern decays, whereas dyad II ends the movement with a constant torque. The (optimal) controllers will never end the movement with wasteful torque, unless they decide to stick to their current position once the task is fulfilled. Looking back at the decoupled controllers (solid traces in Fig 4A), the joint movement ends when the driving controller (blue) has overshot the target and the braking controller (red) is undershooting it. If both controllers maintain their respective positions once their average position reaches the target, the position-priority and velocity-priority controllers will end the movement with constant torque as dyad II did (zoomed plot in Fig 4A). Upon examining the data (Fig 4B), the partner pushing towards the target at movement onset overshot it (overshoot significantly different from zero; one-sample *t*-test on the pushing partner’s position; *t*(15) = 3.73, *p* < 0.002) and the pulling partner undershot the target (*t*(15) = -3.20, *p* < 0.006). Thus, we suspected that pushing at movement onset altered the motion plan to prioritise position, and pulling to prioritise velocity.

We used a Monte Carlo tree-search to identify the underdetermined system of the motion plan priority and final reach position of both partners such that the simulated trajectories and torque pattern matched the data, which were averaged in each block, as closely as possible (see Methods for details). We modified each partner’s state cost matrix **Q** incrementally in the direction that reduced the discrepancy between the simulation and the data. As each pair of controllers yield a unique trajectory and torque pattern, there is no issue in resolving the redundancy while interpreting a torque pattern. Since the trajectory and torque pattern was different between blocks even for the same dyad, we conducted the tree-search for each block separately for every dyad. We could then compare the identified motion plans in the push-pull blocks with those from the coupled block to assess how each partner’s motion plan changed due to their pushing or pulling role in the push-pull block.

**Fig 5:**
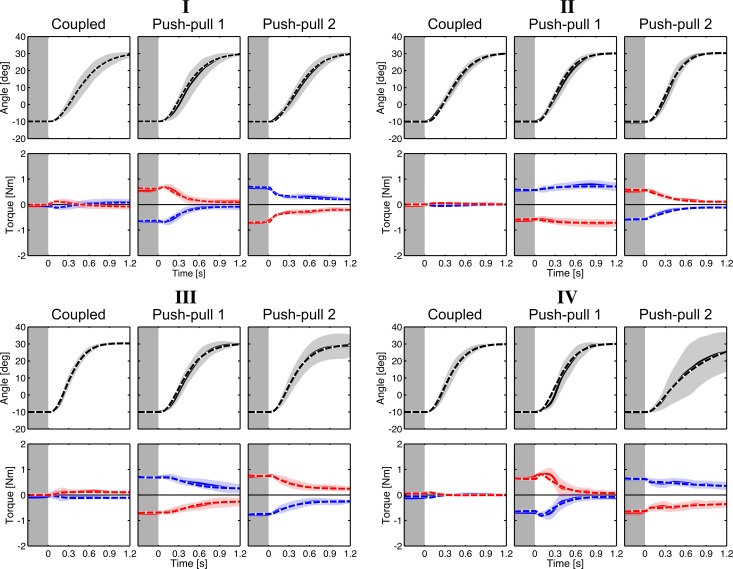
Simulation of dyadic reaching. Trajectories and torques from dyads I to IV (A–D) in the coupled and push-pull blocks. Solid trace is from the data showing the mean of all trials and the shaded area is the 95% confidence interval; the dashed traces are from simulations. First, we identify the state costs of both partners in coupled reaching, then identify the state cost in the push-pull blocks to see what effect the opposing torques prior to movement onset had. In all dyads, the initial opposing torque had a consistent effect: partners pushing towards the target prioritised position, and overshot the target; those pulling away prioritised velocity and undershoot the target.

Fig 5 compares the data (solid trace) and the simulations (dashed trace) of the mean trajectories and torques of all trial from dyads I–IV in both coupled and push-pull conditions. Taking dyad I as an example, in the first coupled block the red partner prioritised position more than blue. This is also evident from the solo blocks where the red partner was found to reach faster than the blue partner (see S2 Table). We simulated the different push-pull conditions using the motion plans identified in the coupled block, but the predicted trajectory and torque were different from the data. Clearly, the partners had different motion plans in the push-pull blocks than in the coupled block. So how had the partners’ motion plan changed? Simulations revealed that in the first push-pull block, the (pushing) red partner prioritised position even more than in the coupled block, and the blue partner prioritised velocity. In the last push-pull block, the (pushing) blue partner prioritised position and the (pulling) red partner prioritised velocity. This pattern of change in the partners' motion plan was consistent in all dyads. Every partner pushing towards the target prioritised position, and those pulling away prioritised velocity (Fig 5B–5D for dyads I–IV, and S2 Fig for dyads V–VIII, see S2 Table for identified state cost terms). This explains why pushing partners, who prioritised position, overshot the target and pulling partners, who prioritised velocity, undershot the target, just as predicted by the computational model (Fig 4A and 4B).

## Discussion

We systematically examined rigidly coupled dyadic reaching movements to observe the trial-by-trial emergence of coordination. We developed a computational model of the reaching movement to identify each partner’s motion plan, thereby resolving the redundancy in interpreting kinematic and torque data. Our experiment revealed that dyads did not change their motion plan, i.e. did not coordinate their behaviour, even after 30 movements. However, their motion plan was different when dyads were constrained to produce a constant opposing torque prior to movement onset. This modification of the motion plan was predictable and dependent on which direction the partner was producing the interaction torque.

Our study provides evidence that dyads do not coordinate during joint reaching movements towards the same target. This result stands in contrast with Reed et al. [2] who argued that the interaction torque was used by dyads to negotiate specialised roles, like one partner who only accelerates and the other who purely decelerates. Indeed, we also observed dyads employing such a ‘strategy’ in our study, but this specialisation can be explained by two reasons. First, partners may reach towards a common target at different speeds or initiate the movement with different reaction times as the authors did not implement a countdown [10]. When we examined the solo blocks where partners were decoupled, the partner who applied torque in the direction of the movement was also reaching faster in the solo trials. Second, the condition at movement onset can affect the specialisation; partners who push towards the target prior to movement onset will tend to keep applying such torque throughout the movement. Reed et al. reported that their dyads were applying such opposing torques whilst waiting for the next target to appear, which explains the specialisation they observed. Thus, the trajectory and torque patterns that these authors documented are the result of different movement speeds, reaction times and varying movement onset conditions.

Our computational model resolved the redundancy in interpreting the trajectory and torque patterns observed during joint reaching. This is in contrast with previous studies [2,6] that employed ad-hoc roles defined by the authors to classify the coordination strategies. Such an approach is limited and may be unwise as differences in natural movement speed may account for differences between dyads [10]. Furthermore, the use of a metric like the magnitude of interaction torque [3] is prone to misinterpreting the results of our study as it would suggest greater coordination in push-pull blocks in comparison to coupled blocks, which is evidently not the case as our computational model suggests. Interestingly, our computational model can be implemented in real-time on a robotic manipulandum to interact with a human partner. Future studies can employ this model to test if a robot partner, whose motion plan is programmed to minimize interaction torque on a trial-by-trial basis, may induce the human partner to do the same. However, our experimental results and computational model suggest that joint reaching may not be a suitable task when examining the emergence of physical coordination, as partners do not update their motion plan on a trial-by-trial basis.

So how should future works study physical coordination and its emergence? Comparing the simulation predictions from co-activity with the experimental results may yield clues as to whether partners coordinate their actions. For example, the study by Ganesh et al. [5], which examined tracking in dyads connected by an elastic band, cannot be explained by co-activity, and suggests a change in one’s behaviour with the physical coupling. Our findings suggest that a joint reaching task is a suitable paradigm to compare with a computational model to identify each partner’s motion plan, but is not sufficient to observe systematic trial-to-trial changes in the motion plans. A continuous task such as a pursuit tracking may be more suitable to examine physical coordination [5,8].

## Supporting Information

S1 Fig Torque patterns from dyads V–VIII in the coupled and push-pull blocks. (EPS)

S2 Fig Measured and predicted trajectories and torque waveforms from dyads V–VIII in the coupled and both push-pull blocks. (EPS)

S3 Fig Example torque difference curves, coupled block, dyad VIII. Example of all pairwise comparisons of torque patterns between bins in the coupled block for dyad VIII. The figures show the *torque difference* Δτ between the mean torque patterns of the bins. Black traces: mean difference curves. Blue traces: statistically significant difference curves identified with wavelet-based single-factor ANOVA. When the blue trace is zero, no significant differences were found between the two bins. Non-zero values indicate significant differences. Red traces: statistically significant difference curves identified with ANOVA performed in the time domain. For dyad VIII, bin 1 showed significantly higher interaction torques compared to other bins (see the torque patterns of dyad VIII in Fig 1). Other comparisons between bins were not significantly different. Statistically significant difference curves identified with ANOVA in the time-domain corroborate the wavelet-domain ANOVA results. S4–S11 Figs show all the torque difference curves as well as the wavelet-based difference curves for all dyads and all connected blocks.
(EPS)

S4 Fig Torque mean difference and wavelet-based ANOVA difference curves, all connected blocks, dyad I. (EPS)

S5 Fig Torque mean difference and wavelet-based ANOVA difference curves, all connected blocks, dyad II. (EPS)

S6 Fig Torque mean difference and wavelet-based ANOVA difference curves, all connected blocks, dyad III. (EPS)

S7 Fig Torque mean difference and wavelet-based ANOVA difference curves, all connected blocks, dyad IV. (EPS)

S8 Fig Torque mean difference and wavelet-based ANOVA difference curves, all connected blocks, dyad V. (EPS)

S9 Fig Torque mean difference and wavelet-based ANOVA difference curves, all connected blocks, dyad VI. (EPS)

S10 Fig Torque mean difference and wavelet-based ANOVA difference curves, all connected blocks, dyad VII. (EPS)

S11 Fig Torque mean difference and wavelet-based ANOVA difference curves, all connected blocks, dyad VIII. (EPS)

S1 File Experiment data file. (ZIP)

S1 Table Summary of significant differences between within-dyad binned torque patterns. Some statistical differences were found between within-dyad binned torque patterns. Significant differences between bins are indicated for each dyad and each coupled and push-pull blocks as follows: first, the bins between which the differences were found are indicated, followed by the time window of the reaching movement in which the differences were found. For example, the significant difference in torque between bins 1 and 2 for dyad VIII in the coupled block (see S1 Fig) would be indicated as: “Bin 1 –Bin 2 (0.68–0.88 s)”. All the significant differences are the result of a significant F tests at a level of significance of 0.05. A dash (–) indicates that no significant differences were found.
(PDF)

S2 Table Summary of identified state cost terms, for all blocks and all dyads. (PDF)
