# Supplementary material for: Motion Plan Changes Predictably in Dyadic Reaching
Source: PLoS One. 2016 Dec 2;11(12):e0167314. doi: 10.1371/journal.pone.0167314 (PMC5135107; doi:10.1371/journal.pone.0167314)
Supplement: S1 Table — Some statistical differences were found between within-dyad binned torque patterns. Significant differences between bins are indicated for each dyad and each coupled and push-pull blocks as follows: first, the bins between which the differences were found are indicated, followed by the time window of the reaching movement in which the differences were found. For example, the significant difference in torque between bins 1 and 2 for dyad VIII in the coupled block (see S1 Fig) would be indicated as: “Bin 1 –Bin 2 (0.68–0.88 s)”. All the significant differences are the result of a significant F tests at a level of significance of 0.05. A dash (–) indicates that no significant differences were found. (PDF) [file pone.0167314.s013.pdf]

Dyad	Coupled	Push-pull 1	Push-pull 2
**I**	–	–	Bin 1 – Bin 5 (0.79 – 1.20 s)<br>Bin 1 – Bin 6 (0.87 – 1.06 s)<br>Bin 2 – Bin 5 (0.88 – 1.20 s)
**II**	–	–	–
**III**	Bin 2 – Bin 3 (0.38 – 0.45 s)	–	–
**IV**	–	–	–
**V**	–	–	–
**VI**	–	–	Bin 2 – Bin 6 (0.78 – 0.90 s) Bin 2 – Bin 6 (0.81 – 1.05 s)
**VII**	–	Bin 2 – Bin 4 (0.70 – 1.2 s)<br>Bin 2 – Bin 5 (0.74 – 1.2 s)	–
**VIII**	Bin 1 – Bin 2 (0.68 – 0.88 s)<br>Bin 1 – Bin 3 (0.57 – 1.04 s)<br>Bin 1 – Bin 4 (0.50 – 1.2 s)<br>Bin 1 – Bin 5 (0.56 – 1.08 s)<br>Bin 1 – Bin 6 (0.69 – 0.86 s)	–	Bin 1 – Bin 4 (0.50 – 0.80, 1.03 – 1.2 s)<br>Bin 1 – Bin 6 (0.50 – 0.85, 0.96 – 1.2 s)
