# Supplementary material for: Motion Plan Changes Predictably in Dyadic Reaching
Source: PLoS One. 2016 Dec 2;11(12):e0167314. doi: 10.1371/journal.pone.0167314 (PMC5135107; doi:10.1371/journal.pone.0167314)
Supplement: S2 Table — (PDF) [file pone.0167314.s014.pdf]

Partner		Coupled		Washout		Push-pull		Washout		Push-pull		Washout	
		Blue	Red	Blue	Red	Blue	Red	Blue	Red	Blue	Red	Blue	Red
Dyad I	Q(1,1)	0.54	2.17	47.17	28.48	0.19	9.01	72.24	27.13	2.58	0.79	103.58	23.75
	Q(2,2)	0.11	0.05	1.46	1.15	0.15	0.21	1.96	1.06	0.05	0.11	1.92	1.22
Dyad II	Q(1,1)	1.91	2.15	26.11	26.28	3.66	5.69	41.54	64.05	1.68	7.5	43.35	63.92
	Q(2,2)	0.13	0.06	1.3	1.33	0.18	0.2	1.58	1.89	0.17	0.02	1.35	1.92
Dyad III	Q(1,1)	7.84	5.56	29.02	49.47	6.84	0.98	21.01	40.83	1.6	11.28	24.94	118.23
	Q(2,2)	0.25	0.15	1.46	1.43	0.01	0.4	1.31	1.47	0.34	0.3	1.42	1.2
Dyad IV	Q(1,1)	3.16	5.13	25.58	26.47	0.3	13.12	113.6	43.74	2.82	0.97	52.63	27.51
	Q(2,2)	0.12	0.22	1.14	1.32	0.23	0.01	1.99	1.6	0.67	1.15	1.75	1.04
Dyad V	Q(1,1)	11.28	8.99	110.79	59.35	4.43	16.11	118.81	152.16	21.41	1.45	58.03	47.89
	Q(2,2)	0.01	0.45	1.7	1.82	0.09	0.57	1.66	2.09	0.01	0.34	1.8	1.45
Dyad VI	Q(1,1)	9.72	6.55	57.35	253.77	16.77	1.28	30.96	127.77	11.12	2.13	24.88	162.88
	Q(2,2)	0.28	0.32	2.21	2.21	0.77	0.13	1.17	2.2	1	0.01	1.41	2.21
Dyad VII	Q(1,1)	2.55	1.9	55.89	22.38	0.3	5.84	22.43	21.88	7.75	0.01	19.27	26.28
	Q(2,2)	0.01	0.19	1.87	1.37	0.01	0.42	1.54	1.74	0.35	0.04	1.98	1.16
Dyad VIII	Q(1,1)	4.3	6.14	42.24	59.92	7.72	1.04	45.77	213.43	1.9	8.76	75.74	61.65
	Q(2,2)	0.23	0.1	1.75	1.74	0.32	0.06	1.4	2.2	0.36	0.01	1.63	1.81
